# The effects of intradermal *M. bovis* and *M. avium* PPD test on immune-related mRNA and miRNA in dermal oedema exudates of water buffaloes (*Bubalus bubalis*)

**DOI:** 10.1007/s11250-021-02696-1

**Published:** 2021-04-06

**Authors:** Carlotta Catozzi, Valentina Zamarian, Gabriele Marziano, Emanuela Dalla Costa, Alessandra Martucciello, Paola Serpe, Domenico Vecchio, Cristina Lecchi, Esterina De Carlo, Fabrizio Ceciliani

**Affiliations:** 1 Department of Veterinary Medicine, Università degli Studi di Milano, Via dell’Università 6, 26900 Lodi, Italy; 2 Istituto Zooprofilattico Sperimentale del Mezzogiorno, National Reference Centre for Hygiene and Technologies of Water Buffalo Farming and Productions, Via delle Calabrie, 27, 84131 Salerno, Italy

**Keywords:** Water buffalo, Tuberculosis, Mycobacterium avium, Mycobacterium bovis, Intradermal reaction, PPD, Immunity

## Abstract

Tuberculosis (TB) is a zoonotic disease primarily caused by pathogens belonging to the genus of *Mycobacterium*. Programs of control and eradication for bovine TB include a screening using single intradermal tuberculin (SIT) test with *Mycobacterium bovis* (*M. bovis*)-purified protein derivatives (PPD-B) single or concurrent with *Mycobacterium avium* (*M. avium*)-purified protein derivatives (PPD-A). This study aimed to determine the effects of intradermal PPD-B and PPD-A test on immune-related mRNA and microRNAs in dermal oedema exudates of water buffaloes (*Bubalus bubalis*). The investigation was carried out on RNA extracted from dermal oedema exudates of 36 animals, of which 24 were *M. bovis* positive (*M. bovis*+) and 12 *M. avium* positive (*M. avium+*). The lymphocyte polarization toward Th1, Th2, TReg, and Th17 lineages was addressed by measuring the abundance of the respective cytokines and transcription factors, namely *TBET*, *STAT4*, *IFNγ*, and *IL1β* for Th1; *STAT5B*, and *IL4* for Th2; *FOXP3* and *IL10* for TReg; and *RORC*, *STAT3*, and *IL17A* for Th17. Due to the very low abundance of Th17-related genes, a digital PCR protocol was also applied. The abundance of microRNAs involved in the immune response against PPDs, including miR-122-5p, miR-148a-3p, miR30a, and miR-455-5p, was equally measured. Results showed that IFNγ (fold change = 2.54; *p* = 0.037) and miR-148a-3p (fold change = 2.54; *p* = 0.03) were upregulated in *M. bovis*+ as compared to *M. avium+* samples. Our preliminary results supported the pivotal role of IFNγ in the local immune response related to PPD-B and highlighted the differential expression of miR-148a-3p, which downregulates the proinflammatory cytokines and the TLR4-mediated NF-κB activation, providing an anti-inflammation modulator in responses to mycobacterial infection.

**Supplementary Information:**

The online version contains supplementary material available at 10.1007/s11250-021-02696-1.

## Introduction

Tuberculosis (TB) is a debilitating zoonotic disease, affecting many domesticated ruminants, including among the others cows (*Bos taurus*) and water buffaloes (*Bubalus bubalis*), caused by pathogens belonging to the genus of Mycobacterium, such as *Mycobacterium bovis* (*M. bovis*), a member of the *Mycobacterium tuberculosis* complex (Pesciaroli et al. 2014). Tuberculosis features the formation of nodular granulomas, most frequently observed in the lymph nodes, lungs, intestines, liver, spleen, pleura, and peritoneum (Palmer and Waters 2006). Screening for TB infection is carried out using the single intradermal tuberculin (SIT) test, based on the inoculation of a *M. bovis*-purified protein derivative (PPD-B). Further tests include in vitro gamma interferon (IFNγ) quantification (Horvat 2015). Diagnosis of TB is finally confirmed by pathology and microbiology after culling. Single SIT test is also applied as a diagnostic test in swamp buffalo (Kanameda et al. 1999) and water buffalo (Javed et al. 2010). In buffalo species, this test is regarded as less sensitive and specific (Kanameda et al. 1999), supposedly due to animal’s malnutrition, which may suppress the test’s response and sensitization with non-tuberculosis mycobacteria. The thickness of the buffalo skin, which is 15–30 mm, as compared to the 6–7 mm of bovine skin, may also interfere with the reaction. To increase specificity, the single intradermal comparative tuberculin test (SICTT), that in water buffaloes has a sensitivity of 71.43% and a specificity of 82.61% (Albernaz et al. 2015), can be carried out with a concurrent inoculation with a *Mycobacterium avium* (*M. avium*)-purified protein derivate (PPD-A) to rule out potential cross-reactivities. *M. avium* is a non-tuberculous Mycobacterium generally present in the environment that can interfere with the *M. bovis* PPD diagnostic test (Horvat 2015). During infection, *M. bovis* is phagocytosed and eliminated by macrophages supported by cytotoxic T (CTL) and type 1 helper T lymphocytes (Th1) (Neill et al. 2001). The polarization toward Th1 lineage is associated with developing a type IV hypersensitivity reaction with the production of cytokines like IFNγ, IL1β, IL12, and TNFα (Winslow et al. 2008; Wang et al. 2011; Lin and Flynn 2015). The *Mycobacterium* may adopt strategies to escape from the immune system at the macrophage level (Zhai et al. 2019) and survive, promoting an evolution toward a chronic disease (Palmer and Waters 2006). During the chronic phase, the CD4^+^ T polarization shifts from type 1 (Th1) to type 2 helper lymphocytes (Th2), promoting humoral immunity and the production of anti-inflammatory cytokines, such as IL4 and IL13. Type 17 (Th17) and regulatory T cells (TReg) are involved in TB immune response (Agrawal et al. 2018) and type IV hypersensitivity reaction (Saini et al. 2018). A recent study determined the gene expression profiling of monocyte-derived macrophages collected from *M. bovis*-infected cattle after in vitro stimulation with *M. bovis* (Shukla et al. 2017). The molecular background of the immune response of water buffalo to intradermal reactions after PPD inoculation is not fully understood. This information is necessary to provide knowledge on the cross-reactivity of *M. bovis* and *M. avium* in infected animals after stimulation with PPDs. This study aims to elucidate the effects of intradermal *M. bovis* and *M. avium* PPD test on immune-related mRNA and miRNA in dermal oedema exudates of water buffaloes (*Bubalus bubalis*) by quantifying the mRNA abundance of transcription factors and cytokines related to Th1, Th2, Th17, and regulatory T cells (TReg) and of four miRNAs (miR-122-5p, miR-148a-3p, miR-30a, and miR-455-5p) associated to immune response and TB.

## Materials and methods

### Identification of animals

Two groups of animals were included in the study:

- *M. bovis* positive (*M. bovis+*): 24 water buffaloes, tested as part of the government prophylaxis program, diagnosed with TB. Animals were positive at single intradermal (SIT) tuberculin tests, single intradermal comparative cervical tuberculin (SICCT), and IFNγ assay. The diagnosis was confirmed for the presence of a tubercular lesion after slaughtering and culture test for *M. bovis*. This group of animals were negative to *M. avium.*
- *M. avium* positive (*M. avium+*): 12 animals that were negative for *M. bovis* and positive for *M. avium* in SICCT.

*M .bovis+* animals were slaughtered following the “The Regional Water Buffalo TB eradication Program”. *M. avium+* samples were collected after slaughtering due to routine culling related to reproductive failure or decreased productive performance, combined with an individual eradication plan for paratuberculosis.

### Diagnosis of TB procedures and exudate collection from dermal oedema

The SIT and SICCT were carried out by intradermal injection of 0.1 ml (30,000 I.U./ml) of PPD-B and 0.2 ml (25,000 I.U./ml) of PPD-A. Both PPD were provided from Istituto Zooprofilattico Sperimentale Umbria e Marche, Italy, following the protocol of “Research project financed by Italian Ministry of Health” and in accordance to the European Community regulations and Italian Legislation: DECREE No 592 of 15 December 1995, LEGISLATIVE DECREE No 196 of 22 May 1999 — Commission Regulation (EC) No 1226/2002, Ministerial Ordinance 9 August 2012 — and subsequent amendments). Both PPD were intradermally injected using Inj-Light tuberculin syringes (18G × 1–1/2, Chemil - Italy). The skin-fold thickness was measured after 72 h with the use of a calliper. The animals were regarded as positive if swelling at the injection site >4 mm (Table S1).

The IFNγ assay was carried out on heparinized blood samples collected from each animal before the SICCT, transported to the laboratory at RT, and co-incubated with avian (PPD-A) (Istituto Zooprofilattico Sperimentale Umbria e Marche, Italy) and bovine (PPD-B) (Thermo Fisher Scientific, Lelystadt, Netherlands). Incubation with phosphate buffer saline (PBS) was used as a negative control, and pokeweed mitogen (Sigma-Merck, Milano, Italy) was included as a positive control. The detection of gamma interferon (IFNγ) was carried out using a commercial assay (BOVIGAM™) (Thermo Fisher Scientific, Schlieren, Switzerland) (Wood and Jones 2001). The samples are regarded as positive for *M. bovis* if both PPD-B were two times higher than the negative control (PBS), or the ratio between PPD-B and PPD-A was ≥1.1.

After slaughtering, the exudate from dermal oedema induced by tuberculin injection was collected using a syringe with a fine needle (size: 18G - 1.20 × 40 mm). The slaughtering of all the animals included in this study was carried out from 1 to 3 days after detecting the local inflammatory reaction. An amount of at least 100 μl was collected from each exudate. RNA later was immediately added to the sample, left overnight at 4 °C, and then stored at a temperature of −80 °C until processing.

Slaughtered animals were subjected to post-mortem examination to detect the presence of TB compatible lesions from retropharyngeal, mandibular, tracheobronchial, mediastinal, mesenteric, hepatic, sub iliac, supra mammary, popliteal, prescapular lymph nodes, spleen, and tonsils. The samples were transported to the laboratory, frozen, and then processed as previously reported (Office International Des Epizooties 2014).

### mRNA and small RNA extraction

mRNA from Th1 (19), Th2 (20), Th17 (21), and TReg (Hougardy et al. 2007) and of four miRNAs (miR-122-5p, miR-148a-3p, miR-30a, and miR-455-5p) associated with immune response and TB (Ueberberg et al. 2014; Albernaz et al. 2015; Ahluwalia et al. 2017; Wu et al. 2017, 2019) were simultaneously extracted using the miRNeasy Micro kit (QIAGEN, Hilden, Germany). Briefly, 1 ml of QIAzol lysis Reagent (QIAGEN, Hilden, Germany) was added to the dermal oedema exudate (100 μl), homogenized, and incubated for 5 min at room temperature. Then, 3.75 μl (final concentration of 25 fmol) of the *Caenorhabditis elegans* miRNA cel-miR-39 (QIAGEN, Hilden, Germany) was introduced as exogenous synthetic spike-in control. The procedure was carried out following the manufacturer’s instructions and mRNA, and small RNAs were eluted in 20 μl of H_2_O for molecular biology.

### mRNA quantification by RT-qPCR

The quality and quantity of recovered RNA were assessed using a NanoDrop ND-1000 UV–vis spectrophotometer (Thermo Fisher Scientific, Massachusetts, USA). A total amount of 1 μg of RNA was treated with DNase (DNase I, RNase-free kit - Fermentas) and reverse transcription (iSCRIPT cDNA Synthesis kit – Bio-Rad, California, USA) in a final volume of 20 μl per each sample. qPCRs were carried out in duplicate for all targets listed in Table 1.

**Table 1:** Sequences of oligonucleotide primers used in the current study and design on the basis of GenBank sequences, except YWHAZ from (26), H3F3A from (27), IL4 from (28), and IL10 from (29)

Target gene; accession number	Sequence		Primer concentration (nM)	Efficiency (%); *R*^2^; Ta (°C)	Amplicon length
TBET XM_006074324.2	Fw 5′➔3′	GCCGTCCCCAGCCTTTTCTGTC	250	94.4%; 0.998; 61.5 °C	170
	Rv 5′➔3′	ACCCACAGCCAGAAGCAGCACC			
STAT4 XM_025277672.1	Fw 5′➔3′	CGTTGGTCGTGGCCTGAACT	300	94.2%; 0.996; 61.5 °C	95
	Rv 5′➔3′	TGGCCCAGGTGAGATGACCA			
IL1B NM_001290898.1	Fw 5′➔3′	AGCTGCATCCAACACCTGGACC	300	99.1%; 0.996; 61.5 °C	110
	Rv 5′➔3′	ACAATGACCGACACCACCTGCC			
IFNG NM_001290905.1	Fw 5′➔3′	GCTCTGCGTGCTTCTGGGTTT	300	109.1%; 0.994; 61.5 °C	117
	Rv 5′➔3′	GGGCCACCCTTAGCTACATCTG			
STAT5B XM_025280120.1	Fw 5′➔3′	TCTCCCCCGACCCCCATTTTCC	250	93.7%; 0.995; 61.5 °C	81
	Rv 5′➔3′	CCACGACTTCCCTTGCCCCAAC			
IL4 AY293620	Fw 5′➔3′	GTACCAGTCACTTCGTCCAT	300	99.2%; 0.990; 52,0 °C 20 s (elongation at 72 °C 25 s)	197
	Rv 5′➔3′	GCTCCTGTAGATACGCCTAA			
FOXP3 XM_006073647.2	Fw 5′➔3′	ACCTGGAAGAATGCCATCCGCC	300	90%; 0.997; 61.5 °C	147
	Rv 5′➔3′	TGTGGGGTTGGAACACCTGCTG			
IL10 AB246351	Fw 5′➔3′	TGCCACAGGCTGAGAACCA	300	97.7%; 0.991; 60 °C	60
	Rv 5′➔3′	TCTCCCCCAGCGAGTTCA			
H3F3A NM_00101489	Fw 5′➔3′	CGCAAACTTCCCTTCCAGCGTC	250	94.3%; 0.995; 61.5 °C	102
	Rv 5′➔3′	TCACTTGCCTCCTGCAAAGCAC			
YWHAV NM_174814	Fw 5′➔3′	GCATCCCACAGACTATTTCC	250	97.3%; 0.998; 61.5 °C	119
	Rv 5′➔3′	GCAAAGACAATGACAGACCA			

Each reaction was composed of 7.5 μl of SsoFast^TM^EvaGreenSupermix (Bio-Rad, California, USA), forward and reverse primers (listed in Table 1), RNase- and DNase-free water, and 1 μl of cDNA with a final volume of 15 μl. The thermal profile consisted of 95 °C for 10 min, 40 cycles of 95 °C for 10s and 60, 61, or 61.5 °C (Table 1) for 30s; the melting curve was assessed by 80 cycles starting from 55 °C with an increase of 0.5 °C each 5 s up to 95 °C. The CFX Connect Real-Time PCR Detection System (Bio-Rad, California, USA) was used to perform the qPCR. Two reference genes (YWHAZ and H3F3A) were selected and the mean of reference gene abundance was used for normalization purposes using the 2^−ΔΔCq^ method. The efficiency of qPCR and *R*^2^ was determined using a relative standard curve (Table 1). Negative controls of qPCR were included by adding nuclease-free water. The Minimum Information for Publication of Quantitative Real-Time PCR (MIQE) guidelines were followed (Bustin et al. 2009).

Digital PCR (dPCR) was carried out to quantify the Th17-related targets, namely *RORC* (Assay ID Bt03256306), *STAT3* (Assay ID Bt01653077), and *IL17A* (Assay ID Bt03210252). *YWHAZ* (Assay ID Bt01122444) was used for data normalization. All probes were checked for identity with the water buffalo genome. A total of 12 samples (6 from *M. bovis*+ and 6 from *M. avium*+) were included based on qPCR results. Each reaction was composed of 1 μl of cDNA, 8 μl QuantStudio 3D Digital PCR Master Mix v2 (Applied Biosystem, California, USA), 0.8 μl of TaqMan Advance (Applied Biosystem), and RNase- and DNase-free water up to 16 μl of the final volume. Fifteen microliters of each reaction was loaded into the chip and run using the QuantStudio 3D Digital PCR System (Thermo Fisher Scientific, Massachusetts, USA). The thermal profile consisted of 95 °C for 10 min, 45 cycles of 60 °C (for *YWHAZ*, *RORC*, and *STAT3*) or 56 °C (for *IL17A*) for 1 min and 98 °C for 30s, followed by 60 °C for 2 min. One negative template control was used for each PCR and then applied to establish the threshold for data analysis performed using the QuantStudio 3D AnalysisSuiteCloud Software.

### Quantification of immune-related miRNA

Two microliters of miRNA was reverse transcribed to cDNA using TaqMan Advanced miRNA cDNA Synthesis Kit (Applied Biosystems, California, USA), following the manufacturer’s procedure. The cel-miR-39 spike-in (Assay ID478326_mir) and four miRNA, namely miR-122-5p (Assay ID 480899), miR-148a-3p (Assay ID 477814), miR-30a (Assay ID 478273), and miR-455-5p (Assay ID 478113), were quantified by qPCR using the Maestro CFX thermocycler (Bio-Rad, California, USA). All probes were checked for identity with the water buffalo genome. Each reaction contained 7.5 μl of 2× TaqMan Fast Advanced Master Mix (Thermo Fisher Scientific, Massachusetts, USA), 0.75 μl of miRNA-specific TaqManAdvance assay (20×) (Thermo Fisher Scientific, Massachusetts, USA), 1 μl of cDNA, and DNase- and RNase-free water up to the final volume of 15 μl. The thermal profile was composed of 50 °C for 2 min, 95 °C for 3 min and 40 cycles of 95 °C for 15 s and 60 °C for 40s. Data normalization was carried out through the spike-in, and miRNA quantification was performed on CFX Maestro™ Software (Bio-Rad, California, USA) using the 2^−ΔΔCq^ method. Negative controls of qPCR were included by adding nuclease-free water.

### Statistical analysis

Statistical analysis was performed using SPSS 23 (SPSS Inc., Chicago, IL, USA) and XLSTAT softwares. Differences were considered to be statistically significant if *p* ≤ 0.05. The data were tested for normality using the Kolmogorov-Smirnov test, while the Levene test was used for testing homogeneity of variance. *TBET*, *IFNγ*, *IL1β*, *STAT5B*, *FOXP3*, *IL10*, *STAT3*, and *IL17A* were not normally distributed, and therefore square root transformation was used. A *T*-test for independent samples was then used to investigate differences between groups (*M. bovis+* and *M. avium+*). Mann-Whitney test was used to investigate whether miR-122-5p, miR-148a-3p, miR30a, and miR-455-5p were differently expressed in the two groups.

## Results

### Quantification of transcription factors and cytokines mRNA related to T cell switching by RT-qPCR

**Fig. 1:**
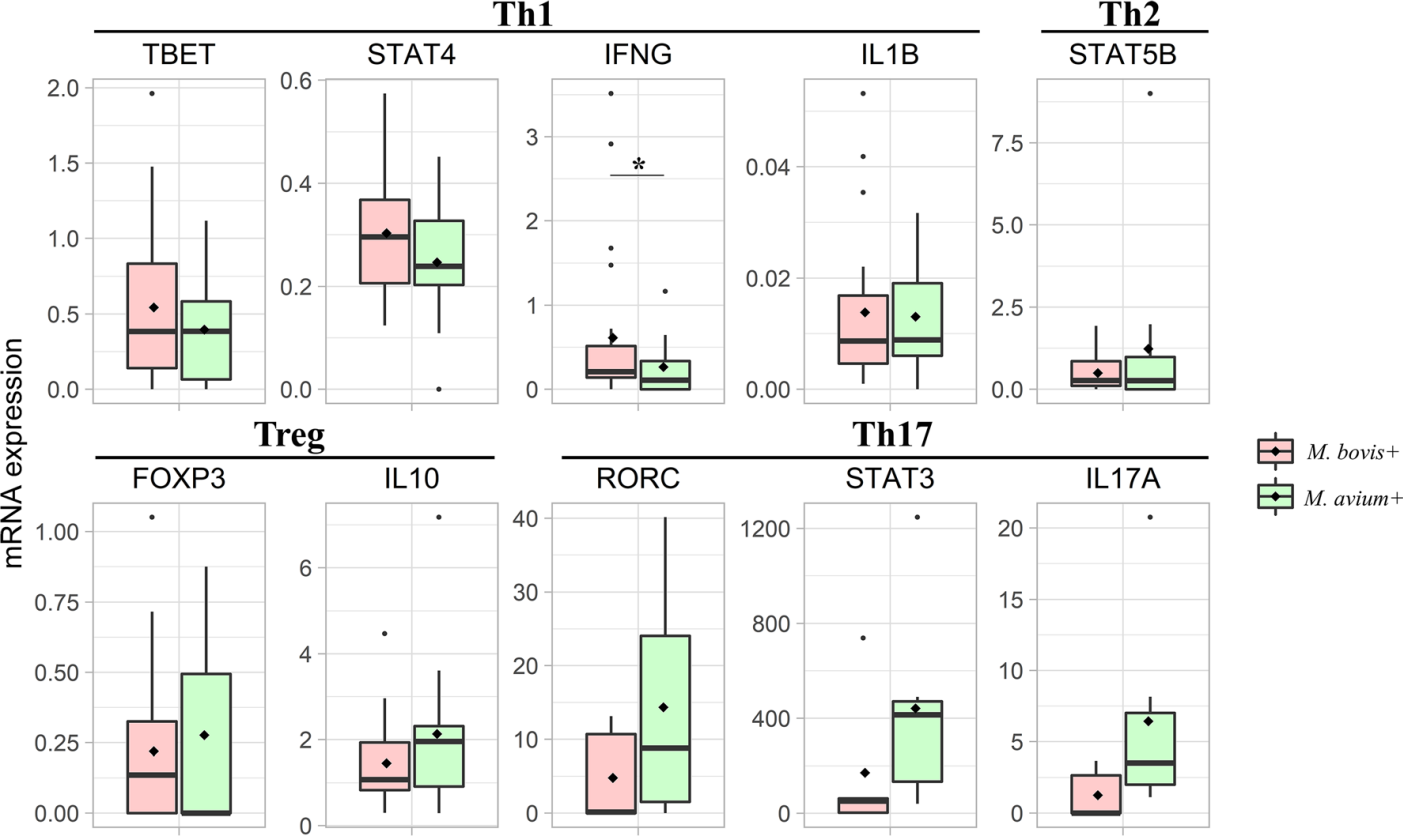
Relative expression of transcription factors and cytokines related to Th1, Th2, TReg, and Th17 polarization. Results for the target genes were normalized using the mean of reference genes (YWHAZ and H3F3A). Data are shown as the mean ± SE of 36 animals for Th1, Th2, and TReg polarization (qPCR) and 12 animals for Th17 polarization (dPCR). Significance was declared for *p < 0.05. The black lines inside the boxes mark the medians. The black diamonds in the boxes mark the mean. Whiskers indicate variability outside the upper and lower quartiles. *M. bovis*+ group is shown in red (n. 24); *M. avium*+ is shown in green (n. 12)

The expression level of 11 targets, including transcription factors and cytokines related to T cell switching, was measured on 36 samples. Since the abundance levels of Th17-related genes were under the limit of detection using conventional RT-qPCR, the analysis was repeated using dPCR. Results are presented in Fig. 1. For Th1-related targets, *M. bovis*+ samples displayed an upregulation of *IFNγ* mRNA (fold change = 2.54; *p* = 0.037) compared to *M. avium*+, whereas no differences were found for *IL1β*, *STAT4*, and *TBET*. For Th2-related targets, the mRNA of IL4 was not detected, and no difference between the expression levels of *STAT5B* between the two groups was observed. For TReg-related targets, *IL10* and *FOXP3* were detected in all samples, but there was no differential expression between *M. bovis*+ and *M. avium*+ animals. For Th17-related targets, quantified using dPCR and TaqMan probes, all targets were detected. Still, no differences were significant, even if a trend of decreased expression of *STAT3* and *IL17A* in *M. bovis+* animals was evident.

### Quantification of immune-related miRNA

**Fig. 2:**
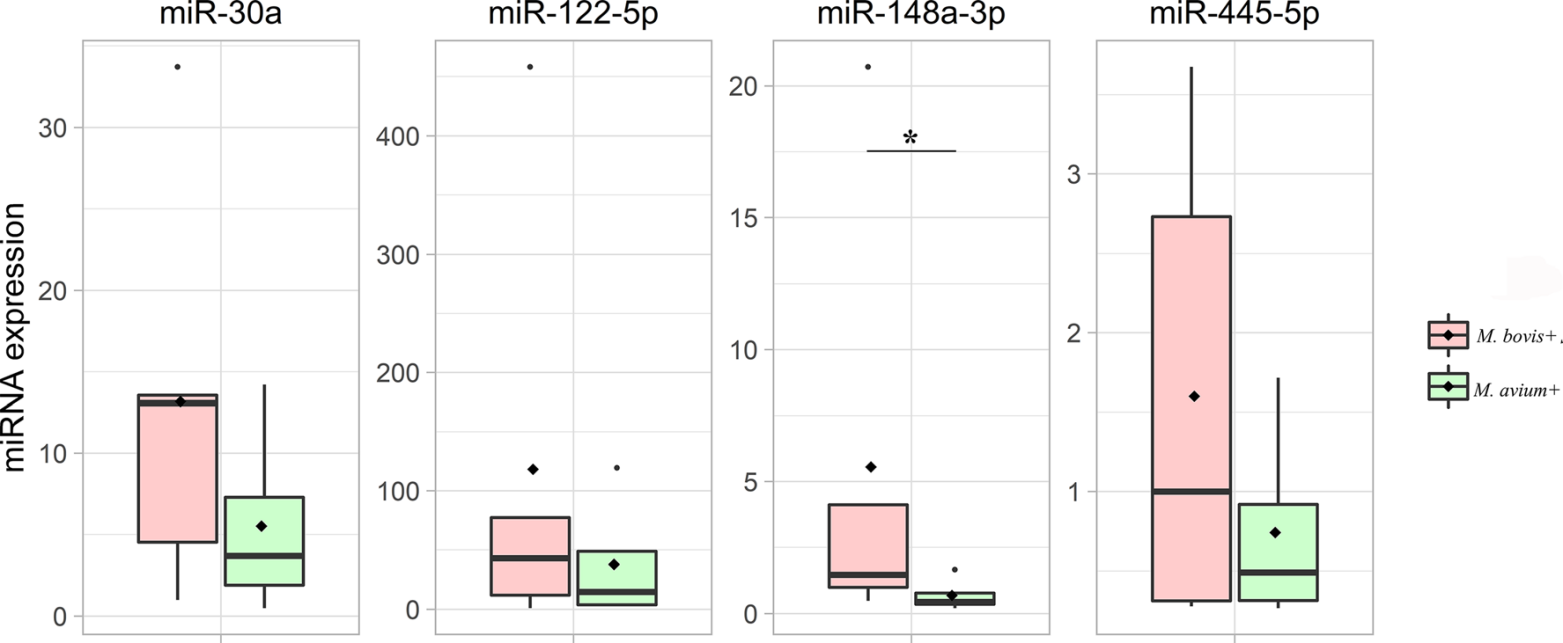
Box plots of immune-related miRNAs. Significance was declared for *p < 0.05. The black lines inside the boxes mark the medians. Whiskers indicate variability outside the upper and lower quartiles. *M. bovis*+ group is shown in red (n. 5); *M. avium*+ is shown in green (n. 4)

Only those samples (n. 9) where the internal control (cel-miR-39) was correctly quantified were considered for the analysis. Results are reported in Fig. 2. Four TB-related miRNAs (miR-122-5p, miR-148a-3p, miR30a, miR-455-5p) were measured in *M. bovis*+ (n.5) and *M. avium*+ (n.4) animals. Although all miRNAs targets were over-expressed in *M. bovis+* animals, only miR-148a-3p was different between the two groups (Mann-Whitney test, *p* = 0.03).

## Discussion

To the best of the authors’ knowledge, this is the first study to investigate at the molecular level the differences in immune-related mRNA and miRNA abundance in dermal oedema exudates of water buffaloes after inoculation of PPD-B (*M. bovis*) and PPD-A (*M. avium*). The study aimed at determining the effects of the two PPD on the intradermal oedema immune reaction, focusing on Th1, Th2, TReg, and Th17 immune response and microRNA involved in the immune response against TB. It was found that the main difference between *M. bovis+* and *M. avium+* dermal oedema molecular milieu was the upregulation of *IFNγ* and miR-148a-3p in *M. bovis*+ dermal oedema. From a methodological perspective, this study provides an effective method to extract and analyze both mRNA and microRNA from the dermal oedema exudate generated by the local injection of PPD-A and PPD-B. This study also provides a protocol to apply digital PCR to local detection of genes related to inflammation in water buffaloes when conventional RT-PCR proves to be not adequately sensitive. The main issues related to the extraction methods are associated with the thickness of the water buffalo skin. Although valid for some samples, in others, the amount of biological material extracted was not enough to obtain results, in particular where the target mRNA was present in a limited amount, such as the Th17-related genes, namely RORC, STAT3, and IL17A. In this case, a digital PCR analysis was carried out, allowing for detecting and measure all the targets. The finding that INFγ mRNA is upregulated is consistent with its pivotal role in immune defence against intracellular pathogens by mediating macrophage activation (Flynn et al. 1993). Increasing IFNγ concentration parallels activation of Th1 immunity in challenged animals compared to the vaccinated ones (Widdison et al. 2006). The high expression of the Th1-associated IFNγ was also observed in polymorphonuclear cells derived from *M. bovis*-infected cows (Blanco et al. 2009). The role of TReg response has been poorly investigated in cattle. In humans, it has been found that TRreg inhibits human memory γδ T cells, reducing the production of IFNγ (Li and Wu 2008) and depressing the T cell-mediated immune response (Hougardy et al. 2007). Our study did not observe any differential expression in Th2 targets between *M. bovis*+ and *M. avium*+ groups, for what concerns TReg and Th17. No differences between *M. bovis* and *M. avium* positive samples were found as well.

The second part of the study measured the differential abundance within the dermal oedema exudates of four immune-related miRNAs, namely mir-122-5p, miR-148a-3p, miR-30a, and miR-455-5p, that were demonstrated to be involved in immune reaction during TB (Ahluwalia et al. 2017; Wu et al. 2017, 2019). Possible issues in miRNA extraction and quantification could be due to the sample matrix. To the best of the authors’ knowledge, the dermal oedema exudate was used as a source to purify miRNA for the first time. Therefore, taking into account the small dataset, results should be considered as preliminary. Only miR-148a-3p was upregulated in a statistically significant way in water buffalos locally injected with *M. bovis* PPD compared to those injected with *M. aviu*m PPD. This finding is consistent with other reports that provided evidence at a systemic level of serum upregulation of miR-148a-3p in TB human patients (Miotto et al. 2013). Remarkably, the systemic upregulation of miR-148a reduces Mycobacterium intracellular survival, and in turn, it is downregulated by the Mycobacterium virulence factor ExsA.

Moreover, upregulation of miR-148a downregulates the proinflammatory cytokines and the TLR4-mediated NF-κB activation, providing an anti-inflammation modulator in responses to mycobacterial infection (Wu et al. 2019). Even if preliminary, the present results might confirm those from a previous study on circulating miRNAs during *M. avium* infection in bovine species that did not detect any change in miRNA-148a abundance, confirming that miR-148a is probably not regulated by *M. avium* infection (Farrell et al. 2015). In humans, the microRNA expression pattern in TB is related to the time and stage of infection (Kleinsteuber et al. 2013) and age (Corral-Fernández et al. 2017). In dairy cows, it has been recently demonstrated that plasma miRNA profiles are related to age and genetic background (Ioannidis et al. 2018), milk production and composition, and the presence of diseases such as mastitis lameness and metabolic stress. Changes in miRNA profile were also found during the dry period and early lactation (Webb et al. 2020). The animals included in this study were clinically healthy, in their third/fourth lactation, and in the mid-lactating period. Moreover, the investigation’s focus was on the local expression profile of miRNA, not in plasma. Still, given how much miRNA profile is related to physiological changes, the health, lactation period, parity, and age of dairy animals should be considered when planning experimental designs involving miRNA analysis.

In conclusion, this study presents a protocol to extract and analyze cytokines and microRNA directly from the inflammatory exudate, providing valuable tools to study at molecular levels the local development of type 4 hypersensitivity. As compared to *M. avium*, the significant finding is that the exudate in *M. bovis*-positive animals presents an upregulation of the Th1-related IFNγ and miR-148a-3p, suggesting the development of type IV hypersensitivity in *M. bovis*-positive animal only. The finding that miR-148a-3p is differentially regulated at the local level within the inflammatory milieu should be validated at a systemic level on a more significant number of cases to identify this miRNA as a potential candidate for differential screening between *M. bovis* and *M. avium* infection.

## Supplementary information

ESM 1 (DOCX 15 kb)
