# Supplementary material for: The effects of intradermal M. bovis and M. avium PPD test on immune-related mRNA and miRNA in dermal oedema exudates of water buffaloes (Bubalus bubalis)
Source: Trop Anim Health Prod. 2021 Apr 6;53(2):250. doi: 10.1007/s11250-021-02696-1 (PMC8024229; doi:10.1007/s11250-021-02696-1)
Supplement: Supplementary file 1 — (DOCX 15 kb) [file 11250_2021_2696_MOESM1_ESM.docx]

Table S1: The skin-fold thickness after intradermal reaction

| **Animal ID** | **Diagnosis** | **SIT PPD-B** | **SIT PPD-B**  **post 72hr** | **SIT PPD-A** | **SIT PPD-A**  **post 72hr** | **SICCT** | **Pathology** |
| --- | --- | --- | --- | --- | --- | --- | --- |
| 9 | M. bovis+ | 17 | 36 | 15,5 | 18,5 | 16 | Y |
| 11 | M. bovis+ | 18 | 39,5 | 16,5 | 25,5 | 12,5 | Y |
| 15 | M. bovis+ | 19 | 24,5 | 21 | 21 | 5,5 | Y |
| 17 | M. bovis+ | 19 | 29 | 18 | 20,5 | 7,5 | Y |
| 19 | M. bovis+ | 20,5 | 35 | 20,5 | 26 | 9 | Y |
| 53 | M. bovis+ | 21 | 32,5 | 21 | 21,5 | 11 | Y |
| 55 | M. bovis+ | 16,5 | 31,5 | 15 | 24 | 6 | Y |
| 57 | M. bovis+ | 21,5 | 34,5 | 15,5 | 23,5 | 5 | Y |
| 59 | M. bovis+ | 15,5 | 35,5 | 18 | 24,5 | 13,5 | Y |
| 61 | M. bovis+ | 18,5 | 28,5 | 16 | 19,5 | 6,5 | Y |
| 63 | M. bovis+ | 16 | 43,5 | 15,5 | 23,5 | 19,5 | Y |
| 65 | M. bovis+ | 16 | 30 | 24,5 | 32 | 6,5 | Y |
| 67 | M. bovis+ | 15,5 | 27 | 15,5 | 20 | 7 | Y |
| 69 | M. bovis+ | 15,5 | 22,5 | 16,5 | 19 | 4,5 | Y |
| 71 | M. bovis+ | 14 | 27,5 | 15 | 22,5 | 6 | Y |
| 73 | M. bovis+ | 15 | 43 | 16 | 25,5 | 19,5 | Y |
| 75 | M. bovis+ | 16,5 | 44,5 | 16,5 | 28 | 7,5 | Y |
| 83 | M. bovis+ | 15,5 | 34 | 16,5 | 24,5 | 11,5 | Y |
| 21 | M. bovis+ | 16,5 | 31,5 | 21,5 | 22 | 14,5 | Y |
| 25 | M. bovis+ | 21 | 30 | 21 | 25 | 5 | Y |
| 33 | M. bovis+ | 18 | 36 | 18 | 20 | 16 | Y |
| 35 | M. bovis+ | 16 | >45 | 16 | 22 |  | Y |
| 77 | M. bovis+ | 25,5 | 40,5 | 22 | 27,5 | 9,5 | Y |
| 1 | M. avium+ | 15 | 15 | 15 | 25 | 10 | N |
| 3 | M. avium+ | 20 | 22 | 20 | 28 | 6 | N |
| 23 | M. avium+ | 19 | 19 | 18 | 26,5 | 8,5 | N |
| 31 | M. avium+ | 15 | 16 | 15,5 | 25,5 | 9 | N |
| 39 | M. avium+ | 15 | 16,5 | 15 | 26,5 | 10 | N |
| 41 | M. avium+ | 18 | 25 | 17 | 32 | 8 | N |
| 43 | M. avium+ | 17,5 | 22 | 16,5 | 29 | 8 | N |
| 47 | M. avium+ | 20 | 23 | 18 | 29 | 8 | N |
| 51 | M. avium+ | 22 | 25,5 | 22 | 37 | 11,5 | N |
| 79 | M. avium+ | 16 | 17 | 16 | 25 | 8 | N |
| 81 | M. avium+ | 20 | 21 | 20 | 28 | 7 | N |
| 93 | M. avium+ | 17 | 17 | 17 | 29 | 12 | N |

Values are expressed in mm
